# Effect of quercetin on steroidogenesis and folliculogenesis in ovary of mice with experimentally-induced polycystic ovarian syndrome

**DOI:** 10.3389/fendo.2023.1153289

**Published:** 2023-08-21

**Authors:** Mohd Zahoor Ul Haq Shah, Vinoy Kumar Shrivastva, Manzoor Ahmad Mir, Wajid Mohammad Sheikh, Mohd Ashraf Ganie, Gulzar Ahmed Rather, Majid Shafi, Showkeen Muzamil Bashir, Mohammad Azam Ansari, Meneerah A. Al-Jafary, Mohammad H. Al-Qhtani, Abdalelgadir Musa Homeida, Ebtesam A. Al-Suhaimi

**Affiliations:** ^1^ Laboratory of Endocrinology, Department of Bioscience Barkatullah University Bhopal, Madhya Pradesh, India; ^2^ Department of Bioresources, University of Kashmir, Srinagar, Jammu and Kashmir, India; ^3^ Biochemistry & Molecular Biology Lab, Division of Veterinary Biochemistry, Faculty of Veterinary Sciences and Animal Husbandry, Sher-e-Kashmir University of Agricultural Sciences and Technology, Srinagar, Jammu and Kashmir, India; ^4^ Department of Endocrinology and Metabolism, Sher-i-Kashmir Institute of Medical Sciences, Srinagar, Jammu and Kashmir, India; ^5^ Department of Biomedical Engineering Sathyabama Institute of Science & Technology, Chennai, Tamil Nadu, India; ^6^ Division of Veterinary Pathology, Faculty of Veterinary Sciences and Animal Husbandry, Sher-e-Kashmir University of Agricultural Sciences and Technology, Srinagar, Jammu and Kashmir, India; ^7^ Department of Epidemic Disease Research, Institute for Research & Medical Consultations (IRMC), Imam Abdulrahman Bin Faisal University, Dammam, Saudi Arabia; ^8^ Biology Department, College of Science and Institute for Research and Medical Consultations (IRMC), Imam Abdulrahman Bin Faisal University, Dammam, Saudi Arabia; ^9^ Department of Paediatrics, College of Medicine, Imam Abdulrahman Bin Faisal University, Dammam, Saudi Arabia; ^10^ Department of Environmental Health Research, Institute for Research & Medical Consultations (IRMC), Imam Abdulrahman Bin Faisal University, Dammam, Saudi Arabia

**Keywords:** PCOS (polycystic ovarian syndrome), steroidogenesis, folliculogenesis, quercetin, VEGF

## Abstract

**Introduction:**

Polycystic Ovary syndrome (PCOS) affects the health of many women around theworld. Apart from fundamental metabolic problems connected to PCOS, focus of our study is on the role of quercetin on genes relevant to steroidogenesis and folliculogenesis.

**Methods:**

Eighteen mature parkes strain mice (4-5 weeks old) weighing18–21 g were randomly divided into three groups of six each as follows: Group I serves as the control and was given water and a regular chow diet ad lib for 66 days; group II was given oral gavage administration of letrozole (LETZ) (6 mg/kgbw) for 21 days to induce PCOS and was left untreated for 45 days; For three weeks, Group III received oral gavage dose of LETZ (6 mg/kg), after which it received Quercetin (QUER) (125 mg/kg bw orally daily) for 45 days.

**Results:**

In our study we observed that mice with PCOS had irregular estrous cycle with increased LH/FSH ratio, decreased estrogen level and decline in expression of Kitl, Bmp1, Cyp11a1, Cyp19a1, Ar, lhr, Fshr and Esr1 in ovary. Moreover, we observed increase in the expression of CYP17a1, as well as increase in cholesterol, triglycerides, testosterone, vascular endothelial growth factor VEGF and insulin levels. All these changes were reversed after the administration of quercetin in PCOS mice.

**Discussion:**

Quercetin treatment reversed the molecular, functional and morphological abnormalities brought on due to letrozole in pathological and physiological setting, particularly the issues of reproduction connected to PCOS. Quercetin doesn’t act locally only but it acts systematically as it works on Pituitary (LH/FSH)- Ovary (gonad hormones) axis. the Side effects of Quercetin have to be targeted in future researches. Quercetin may act as a promising candidate for medical management of human PCOS.

## 1 Introduction

Polycystic Ovary syndrome (PCOS) affects the health of many women around the world. Patients with PCOS are typically females in their reproductive years that have one or more of the following conditions: (A) obesity; (B) an irregular estrus cycle (C) sub/infertility or (D) hirsutism. Ovarian dysfunction, cysts in ovaries, and hyperandrogenism are some of its diagnostic markers. Despite the lack of a clear aetiology, it appears an imbalance of hormones, particularly elevated testosterone level, as well as insulin resistance (IR), can be taken into account (1–3). Patients with PCOS who have infertility are frequently upset about their inability to get pregnant. The hypothalamic-pituitary-ovarian axis is hypothesised to be impacted by the environment and genetics in around three and a half of PCOS patients who have elevated androgen levels (4). One of the intraovarian steroidogenesis abnormalities that are hypothesised to lead to ovarian failure in PCOS is a decrease in activity of aromatase enzyme, causing an imbalance of hormones, hyperandrogenism, and excess androgens within the ovaries leading to polycystic ovaries could be anticipated from decreased activity of the enzyme aromatase, which determines production rate of production of estrogen from androgen (3, 5).

As was already mentioned, a significant factor in PCOS is hyperandrogenism (6). In ovarian follicle granulosa cells, aromatase (*Cyp19a1*) changes testosterone (Testo) into oestrogen. As a way to create a model of PCOS having similar features of women with PCOS, we administered letrozole (LETZ) to female mice. LETZ is a a non-steroidal inhibitor of aromatase which results in accumulation of androgen by decreasing the activity of aromatase, thereby lowers production of estrogen (7, 8).

Today, a variety of techniques are employed to combat PCOS and promote ovulation. However, a number of serious side effects, such as arthritis and joint or muscular pain have been observed (9). Consequently, natural medicines having no or few side effect are becoming more and more popular. A flavonoid molecule called quercetin (QUER) has biological properties that include, controlling blood lipid levels, controlling blood sugar and scavenging oxygen free radicals. Its molecular formula is C15H10O7, and its chemical name is 4h-1-benzopyran-4-one, 2-(3,4-dihydroxy phenyl), 3,5,7-trihydroxy-flavone. According to recent research, quercetin can boost healthy ovarian follicle development, restore healthy anatomy of ovary, as well as enhance histology of uterus. It’s effects are comparable to those of metformin (10). According to reports, QUER lowers level of LH, and testo in PCOS patients (11). According to the most recent studies, quercetin can impact ovarian development and possesses estrogen-like effects (12) Researchers also discovered that quercetin can reduce insulin resistance, treat hyperinsulinemia, lower blood sugar levels, and block the expression of androgens (13). It has been observed that Oral QUER supplementation was effective in improving the adiponectin-mediated insulin resistance and hormonal profile of women with PCOS. It has been noted that taking oral QUER supplements helped women with PCOS with their adiponectin-mediated insulin resistance and hormonal profile (14). It has been demonstrated that quercetin lowers ovarian Bax and raises Bcl-2 protein abundance in PCOS rodents. Our findings suggest that QUER may increase oestrogen concentration, ovarian aromatase protein content, folliculogenesis, and decrease atresia by attenuating hyperandrogenism in PCOS rats. QUER is as effective as metformin in reducing hyperandrogenism by lowering free Testosterone level and improving hypothalamic-pituitary-ovarian axis function. (15).

However, despite the fact that some researches have looked at the connection between QUER and PCOS, however they solely looked into impact of QUER on common signs of PCOS. Apart from fundamental metabolic problems connected to PCOS, focus of our study is on the role of quercetin on genes relevant to steroidogenesis and folliculogenesis.

## 2 Materials and methods

### 2.1 Chemicals

Sun Pharma Company and Sigma Aldrich were used to obtain the drugs letrozole and quercetin, respectively. The ELISA kits (ELK Biotechnology Wuhan, China) were bought from Clementia Biotech, New Delhi, India for the hormonal analysis. Analytical-grade chemicals were used in addition during the investigation.

### 2.2 Animals

Eighteen mature parkes strain mice (Age: 4-5 weeks) weighing 18-21 g were procured from Jeeva life sciences Hyderabad, mice having unrestricted access to water as well as food, and we gave them two weeks to acclimatize the environment. Following the acclimation period of two weeks, the animals were randomly into three groups of six each as follows: Group I serves as the control and was given water and a regular chow diet *ad lib* for 66 days; group II was given oral gavage administration of letrozole (LETZ) (6 mg/kg bw) (3) for 21 days to induce PCOS and was left untreated for 45 days; For three weeks, Group III received oral gavage dose of LETZ (6 mg/kg), after which it received Quercetin (QUER) (125 mg/kg bw orally daily) for 45 days. We kept mice under normal ambient temperature (22-25˚C), relative humidity of (55-60) and twelve hours of dark and light cycles respectively. A 50 mg/kg intraperitoneal dose of sodium pentobarbital was used to anaesthetize the mice and by puncturing the retro-orbital venous sinus, blood samples from all the mice were obtained at 66^th^ day of the experiment, and serum was obtained which was then used to analyse hormones and biochemistry. A cervical dislocation was then used to kill the mice. The body had its ovaries removed and adipose tissues were cleansed for further biochemical and gene expression studies. The ethical committee of institution (Barkatullah University Bhopal) gave their consent under 1885/GO/Re/S/CPCSEA/IAEC/BU/21 to all experimental protocols.

### 2.3 Cholesterol and triglyceride analysis

Using easily accessible kits, triglycerides (TG) and total cholesterol (TC) were colorimetrically measured (Meril Diagnostics, Gujrat, India). Indirect measurements of low-density lipoprotein and very low-density lipoprotein were made while using Friedewald’s equation.

Friedwald Equation: LDL = TC – HDL − (TG/5). VLDL= VLDL = TG/5 (16).

### 2.4 Analysis of hormones

The 67th day of the trial saw the collection of blood via retro-orbital venous sinus puncture. A centrifugation process was used to separate the serum, which was then put in storage until it was needed. The Enzyme-linked- Immunosorbent Assay ELISA kits that were used were obtained from ELK biotechnology, Wuhan, China, (CAT. numbers: ELK 368, ELK4808, ELK8407) and were built on the competitive inhibition enzyme immunoassay methodology. The kits’ microtiter plate already has a specific protein pre-coated on it. An anti-testosterone, anti-LH, anti-FSH, and anti-oestrogen antibody biotin-conjugated was added to the appropriate microplate wells once the addition was made of standards or samples. The TMB substrate solution was then added to each microplate well, followed by the addition of an avidin-horseradish peroxidase (HRP) conjugate, which was then incubated for 45 minutes. The enzyme substrate reaction was stopped using the kits’ stop solution, and the colour shift was detected using an ELISA reader that operates at a wavelength of 450 ± 10 nm.

### 2.5 Test procedure for vascular endothelial growth factor in ovarian tissue

The sandwich-ELISA method was used in the ELISA kit purchased from Elabscience Biotechnology Inc., (Wuhan, Hubei, P.R.C., China; CAT. No: E-EL-H0111; intra and inter –CV are <10%) This kit came with a micro-ELISA plate (MEP) that was already coated with a vascular endothelial growth factor-specific antibody (VEGF). The wells of MEP comprised the particular antibody additionally to norms or examples. Then serial injections of an Avidin-Horseradish Peroxidase (HRP) solution and biotin - conjugated antibodies unique to VEGF were produced into each microplate well. The free bits were removed during washing. Each well received a dose of the substrate solution. Colour blue was only visible in the wells containing VEGF, biotinylated detecting antibody, and Avidin-HRP conjugate. Stop solution is added to stop the enzyme-substrate reaction was halted and the colour changed to yellow. At 450 nm, the optical density (OD) was measured using an ELISA reader. The OD value and VEGF levels are linearly correlated. Based on the OD of the data in respect to the conventional curves, the amount of VEGF present for each sample may be determined.

### 2.6 Reverse transcription and real-time PCR

Total RNA was extracted using Invitrogen’s TRI Reagent, and 1µg of total RNA was used to create cDNA, both in accordance with the manufacturer’s instructions (Invitrogen). The RT-PCR was performed using SYBR green and real-time PCR. To calculate the mRNA values, a standard curve for every gene’s related expression level was developed. The SDS software was used to determine the threshold cycle (CT) of each sample and average CT was calculated for triplicate. Moreover Δ CT for each target gene was calculated by subtracting Δ CT for β-actin from average CT of the target gene of the sample. **Table 1** lists the primers and internal controls that were employed, with additional data.

**Table 1:** Description for RT-PCR primers.

Gene	Forward primer	Reverse primer
*Kitl*	GGTAGCCAGGAGTTTGTTCT	TTGTGTGGCATAAGGGCT
*CYP11a1*	TCCTCAAAGCCAGCATCA	ATCTCGACCCATGGCAAA
*CYP19a1*	ATGTTCTTGGAAATGCTGAACCC	AGGACCTGGTATGAAGACGAG
*Bmp1*	GATATTGAGTCTCAGCCCGA	AACATGCGGTTGCCTGTA
*fshr*	CTCATCAAGCGACACCAAGA	GGAAAGGATTGGCACAAGAA
*lhr*	ACACTGCCCTCCAAAGAAAA	CCTCAAAGATGGCGGAATAA
*Ar*	CTGGGAAGGGTCTACCCAC	GGTGCTATGTTAGCGGCCTC
*Esr1*	GAA GGC TGC AAG GCT TTC TT	TCT TTT CGT ATC CCG CCT TT
*CYP17a1*	GCC CAA GTC AAA GAC ACC TAA T	GTA CCC AGG CGA AGA GAA TAG A
*B Actin*	TACGTCGCCCTGGATTTT	ATGAAAGAGGGCTGGAAGAG

### 2.7 Histological assessment of ovaries

Ovarian tissue was fixated in 10% formol-saline for 24 hrs, dried, covered with paraffin, and sections were cut at a thickness of 5 microns for hematoxylin and eosin (H & E) stains. Motic microscope was used to examine and evaluate the sections (17).

### 2.8 Statistical analysis

A one-way analysis of variance as well as a *post hoc* evaluation utilizing Tukey’s multiple comparison tests was utilised to decide the parameters’ importance by using Graph Pad Prism version 9.3. Significance levels of 0.05, 0.01 and 0.001 accordingly have been used to indicate the statistically important, highly remarkable, and extremely significant level.

## 3 Results

### 3.1 Effects of quercetin treatment on levels of insulin in mice with letrozole-induced polycystic ovary

**Figure 1:**
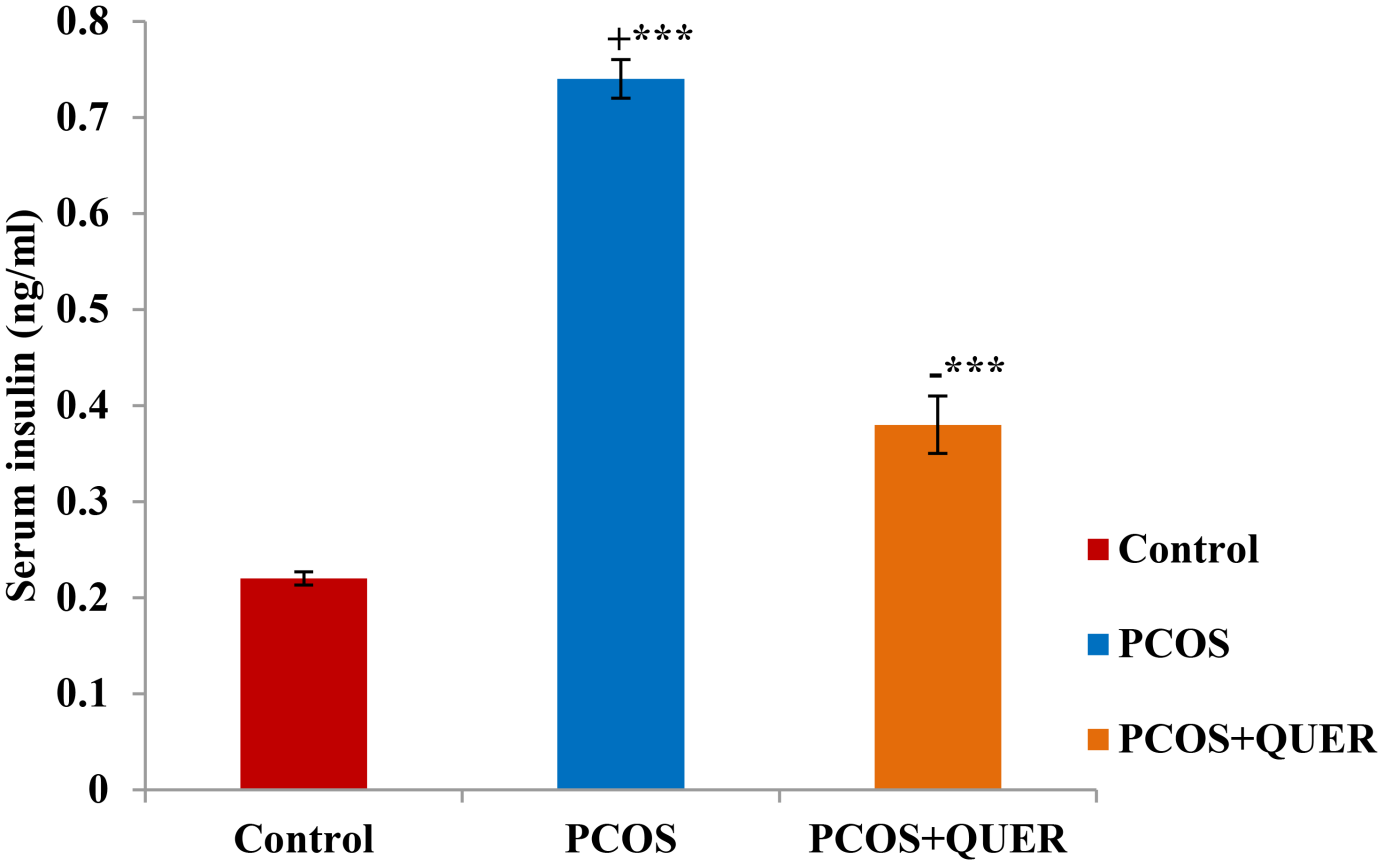
Effect of QUER on Serum insulin in mice with PCOS: With n=6 per group, all values are expressed as means with standard errors: Control: saline solution at 0.9%: PCOS (6 mg/kg of LETZ); PCOS+ QUER (6 mg/kg of LETZ and 125 mg/kg of QUER); += Control versus PCOS; -= PCOS versus PCOS+QUER; ***p 0.001.

Letrozole (LTZ) delivery in this trial caused a substantial rise in blood insulin levels (p 0.001) against healthy controls, and we also discovered a significant fall in insulin in the PCOS group receiving QUER in comparison to the PCOS group not receiving treatment (**Figure 1**).

### 3.2 Effect of quercetin treatment on estrous cycle in mice with letrozole-induced polycystic ovary

The estrouscyclicity of the LETZ-induced PCOS mice was abnormal, and we saw a persistent diestrus condition that led to longer diestrous cycles than in the normal control mice. However, the estrous cycle was regularised after taking QUER, which returned the cycle duration to normal (**Table 2**).

**Table 2:** Effect of QUER therapy on estrous cycle, cholesterol and triglycerides PCOS mice.

Group	Cholesterol	Triglycerides	Average number of cycles in 30 days
**Control**	114.93 ± 1.01	119.76 ± 0.71	5.00 ± 0.31
**PCOS**	144.86 ± 1.95^+***^	141.91 ± 2.49^+***^	1.66 ± 0.21^+***^
**PCOS+QUER**	124.71 ± 1.68^-***^	131.55 ± 2.35^-**^	4.00 ± 0.36^-***^

With n=6 per group, all values are expressed as means with standard errors: Control: saline solution at 0.9%: PCOS (6 mg/kg of LETZ); PCOS+ QUER (6 mg/kg of LETZ and 125 mg/kg of QUER); += Control versus PCOS; -= PCOS versus PCOS+QUER; ***p 0.001, **p 0.01.

### 3.3 Effect of oral quercetin treatment on serum cholesterol and TG levels in mice with letrozole-induced polycystic ovary

When compared to the normal control mice, we saw that LETZ-induced PCOS mice had significantly higher levels of TG and cholesterol (P<0.001). In contrast, we saw that the PCOS group that received QUER had significantly lower levels of TG and cholesterol (P<0.001).

### 3.4 Effect of quercetin on serum hormones in mice with letrozole-Induced polycystic ovary

**Figure 2:**
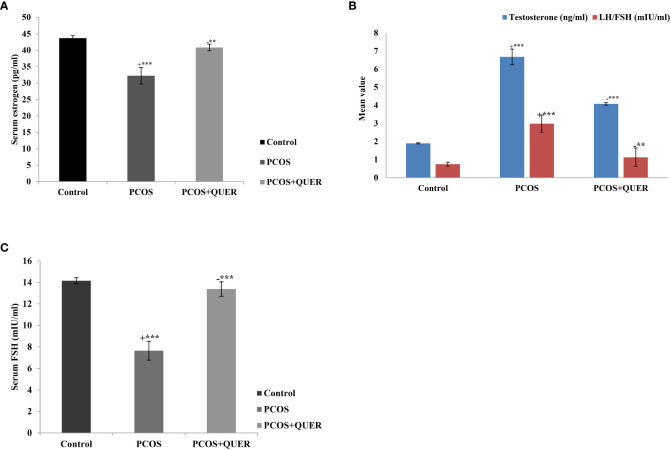
**(A)** Effect of QUER on Serum estrogen in mice with PCOS With n=6 per group, all values are expressed as means with standard errors: Control: saline solution at 0.9%: PCOS (6 mg/kg of LETZ); PCOS+ QUER (6 mg/kg of LETZ and 125 mg/kg of QUER); += Control versus PCOS; -= PCOS versus PCOS+QUER; ***p 0.001, **p 0.01. **(B)** Effect of QUER on LH/FSH and testosterone in PCOS mice. With n=6 per group, all values are expressed as means with standard errors: Control: saline solution at 0.9%: PCOS (6 mg/kg of LETZ); PCOS+ QUER (6 mg/kg of LETZ and 125 mg/kg of QUER); += Control versus PCOS; -= PCOS versus PCOS+QUER; ***p 0.001, **p 0.01. **(C)** Effect of QUER on serum FSH in PCOS mice. With n=6 per group, all values are expressed as means with standard errors: Control: saline solution at 0.9%: PCOS (6 mg/kg of LETZ); PCOS+ QUER (6 mg/kg of LETZ and 125 mg/kg of QUER); += Control versus PCOS; -= PCOS versus PCOS+QUER; ***p 0.001.

We found that, in contrast normal control group, LETZ induced PCOS mice had significantly elevated levels of blood testosterone and LH : FSH ratio and significantly lower serum estrogen and FSH levels. However, QUER treatment significantly reduced (p<0.001) the testosterone as well as LH : FSH levels in PCOS mice, while increasing (p<0.01) the levels of estrogen and FSH compared with the PCOS group that isn’t receiving treatment (**Figure 2**).

### 3.5 Effects of oral quercetin treatment on plasma vascular endothelial growth factor in mice with LTZ Induced polycystic ovary

**Figure 3:**
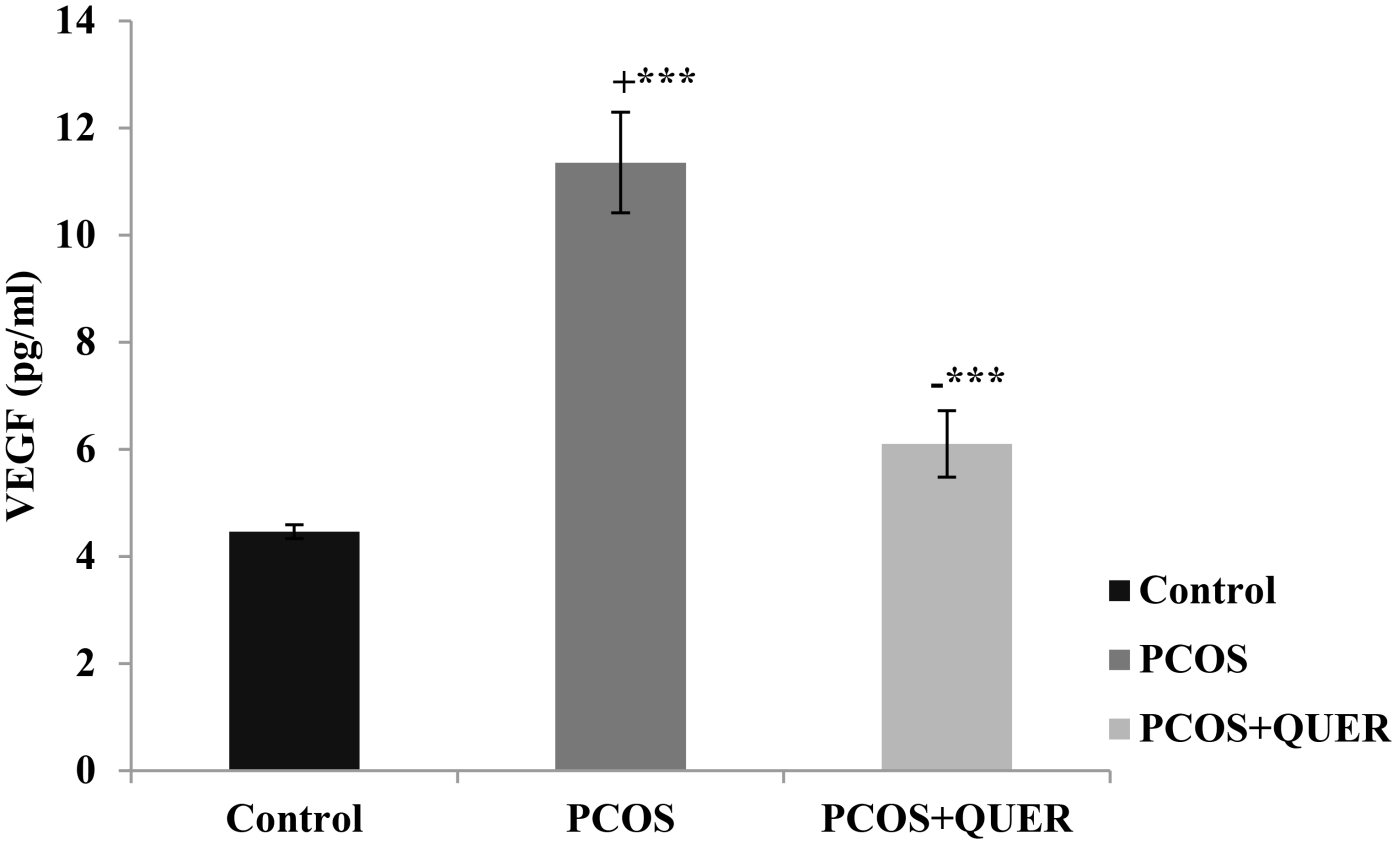
Effect of QUER on plasma VEGF in PCOS mice. With n=6 per group, all values are expressed as means with standard errors: Control: saline solution at 0.9%: PCOS (6 mg/kg of LETZ); PCOS+ QUER (6 mg/kg of LETZ and 125 mg/kg of QUER); += Control versus PCOS; -= PCOS versus PCOS+QUER; ***p 0.001.

This study’s findings demonstrated that when letrozole was administered, VEGF levels significantly increased (p<0.001) when compared to the normal mice. However, in contrast to the LETZ-induced PCOS group, we noticed a significant (P<0.001) drop in VEGF levels in the group that received LETZ + QUER (**Figure 3**).

### 3.6 Effects of oral administration of QUER expression of genes related to folliculogenesis in LETZ induced PCOS mice

**Figure 4:**
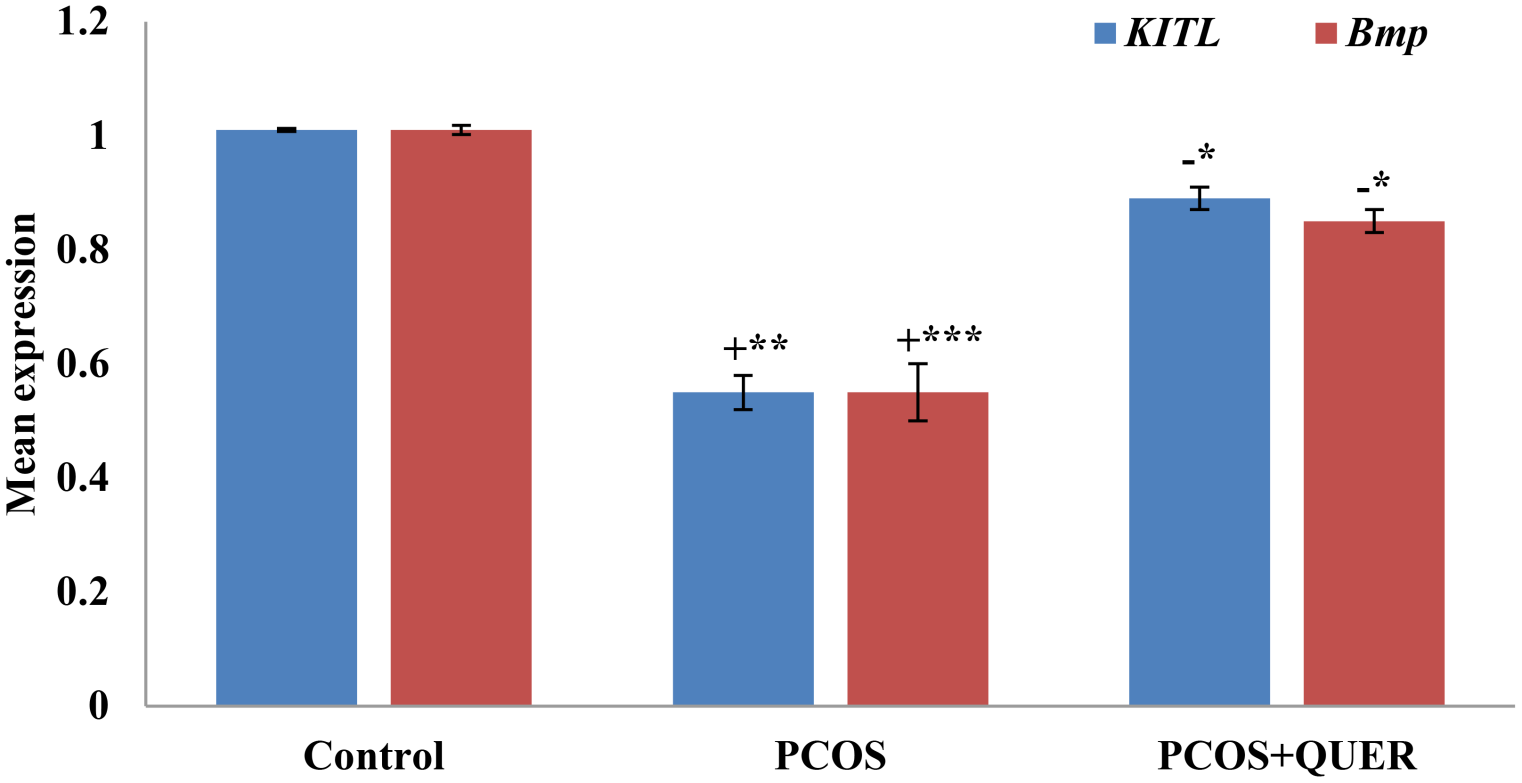
Effect of QUER on *KITL* and *Bmp1* in mice with polycystic ovary syndrome: With n=6 per group, all values are expressed as means with standard errors: Control: saline solution at 0.9%: PCOS (6 mg/kg of LETZ); PCOS+ QUER (6 mg/kg of LETZ and 125 mg/kg of QUER); += Control versus PCOS; -= PCOS versus PCOS+QUER; ***p 0.001, **p 0.01, *p <0.05.

Our findings demonstrated that, in comparison to the usual control, the expressions of *KITL* and *Bmp1* significantly decreased after LETZ treatment (p<0.001). The group that received LETZ + QUER, however, had levels that were importantly higher (p<0.05) in contrast to the mice with PCOS induced by letrozole (**Figure 4**).

### 3.7 Effect of oral QUER treatment on expression of genes related to generation of steroids in mice with LETZ induced PCOS

**Figure 5:**
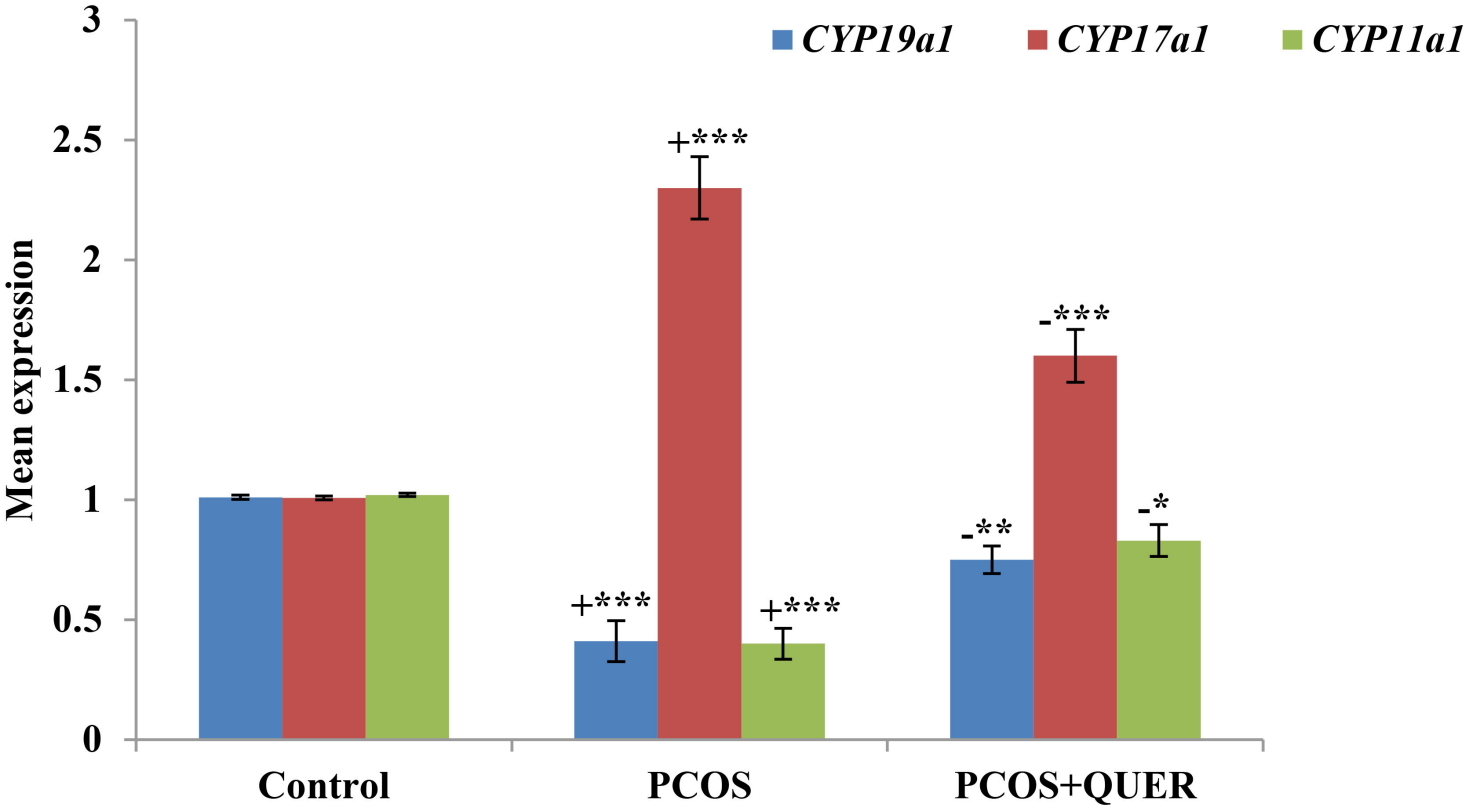
Effects of QUER on *CYP19A1*, *CYP17A1* & *CYP11a1* in mice with PCOS. With n=6 per group, all values are expressed as means with standard errors: Control: saline solution at 0.9%: PCOS (6 mg/kg of LETZ); PCOS+ QUER (6 mg/kg of LETZ and 125 mg/kg of QUER); += Control versus PCOS; -= PCOS versus PCOS+QUER; *p<0.05, **p<0.01,***p<0.001.

The findings demonstrated that mice with PCOS induced by LETZ had considerably increased levels of *CYP17a1* expression and low levels of *CYP19a1* and *CYP11a1* than control mice. *CYP17a1* expression in LETZ + QUER group, however, were significantly lower with the increase in *CYP19a1* and *CYP11a1* (p<0.01) than mice with LTZ induced PCOS (**Figure 5**).

### 3.8 Effect of oral treatment of QUER on expression of receptors of hormones in mice with LTZ induced PCOS

**Figure 6:**
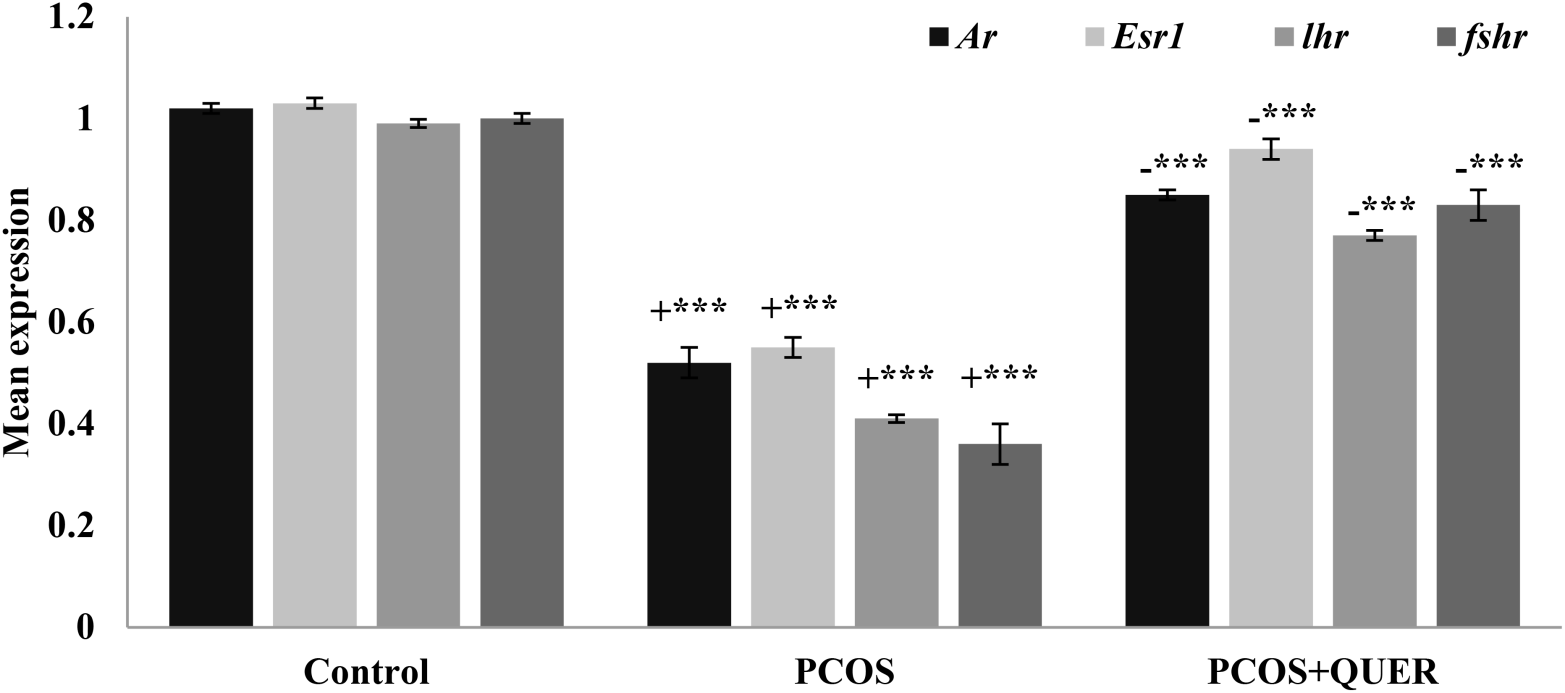
Effects of QUER treatment on *Ar*, *Esr1*, *lhr* and *fshr* in mice with PCOS. With n=6 per group, all values are expressed as means with standard errors: Control: saline solution at 0.9%: PCOS (6 mg/kg of LETZ); PCOS+ QUER (6 mg/kg of LETZ and 125 mg/kg of QUER); += Control versus PCOS; -= PCOS versus PCOS+QUER; ***p 0.001, **p 0.01.

The findings demonstrated that *Ar*, *lhr*, *esr1*, and *fshr* expressions significantly decreased mice with PCOS induced by LETZ (p<0.001). However, we saw a substantial reduction (p<0.01) in the levels of *Ar*, *lhr*, *esr1*, and *fshr* in the LETZ + QUER group in contrast to the PCOS group subjected to LETZ (**Figure 6**).

### 3.9 Effects of oral QUER treatment on ovarian histology in mice with LETZ induced PCOS

**Figure 7:**
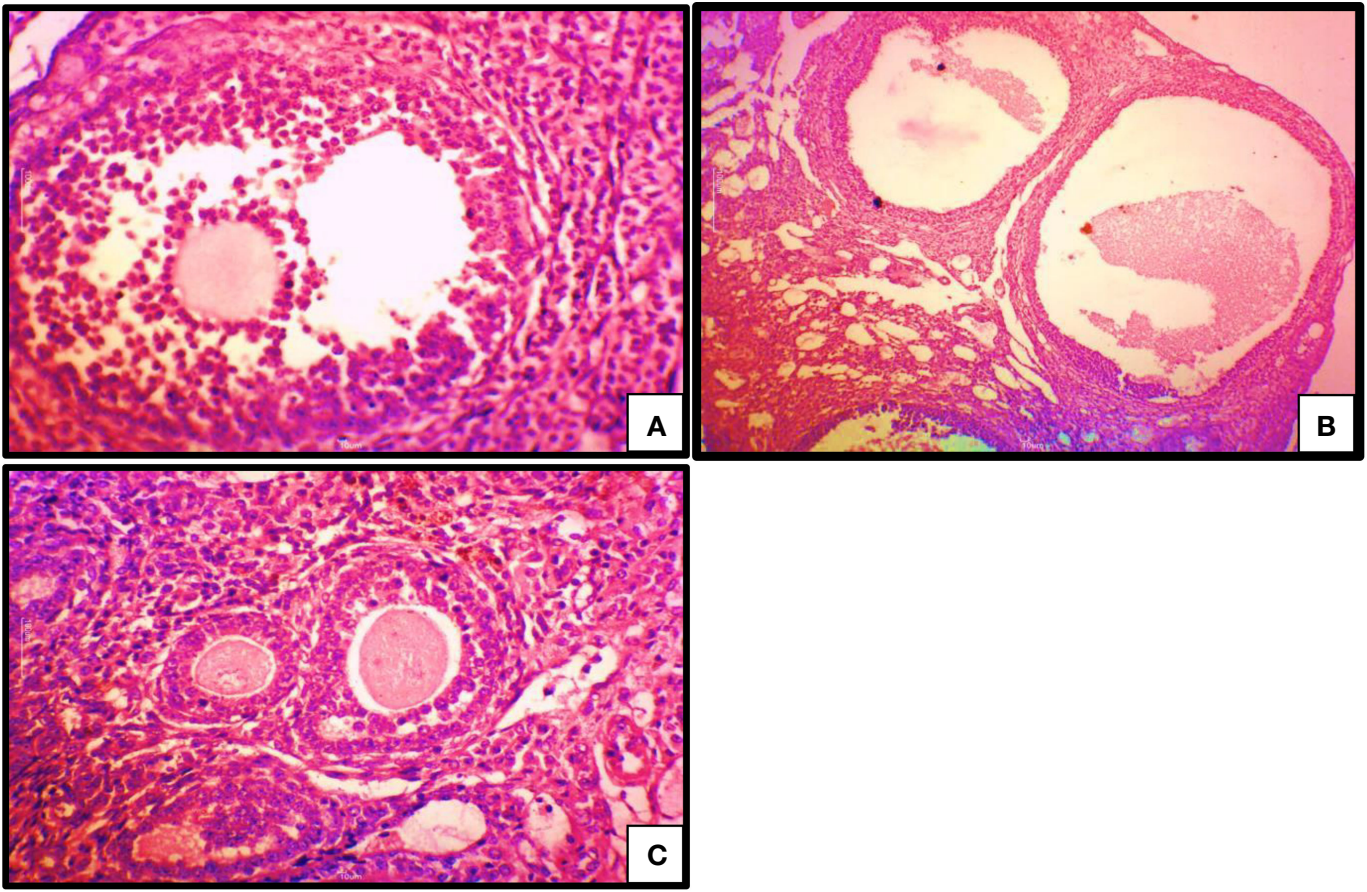
Photomicrographs of histological sections of ovary (H&E, magnificent x50): **(A)** PCOS; showing cystic follicles with degeneration of ovum **(B)** control; showing healthy follicle and ovum **(C)** PCOS treated with QUER; showing regenerated ovum with absence of cystic follicles.

Contrary to the normal control, we saw that letrozole treatment caused the corpus luteum to degenerate, producing more cystic follicles and no ovum. We saw a drop in cystic follicles and a regeneration of the corpus luteum and ovum in the group that underwent oral gavage treatment with LETZ+QUER in contrast to the mice with LETZ induced PCOS (**Figure 7**).

## 4 Discussion

We show here that quercetin (QUER) therapy is linked to anti-androgenic and anti- angiogenesis effects in the mouse ovary using a mice model of PCOS. We further show that the prolonged advantage of QUER improves ovarian function by increasing the control of genes involved to steroidogenesis and folliculogenesis. We demonstrate how QUER alters the functional, molecular, and morphological abnormalities caused by letrozole in pathological and physiological settings, particularly the problems with reproduction associated with PCOS, by regulating steroidogenesis and folliculogenesis in addition to regulating hormone receptors. The estrous cycle is known to cause changes in the hormones that control the activity of the ovaries, including follicle maturation (18). Prolonged estrus cycle with a continuous dioestrus phase was seen in our PCOS mice model, but the deficiencies were alleviated by giving the mice QUER. The PCOS rats also showed an extended diestrus phase and an irregular estrus cycle (19).

Other than type 2 diabetes, which has been previously established, women with polycystic ovarian syndrome also exhibit IR, impaired glucose tolerance, and obesity (20, 21). Result from this supported earlier research in that LETZ-induced PCOS in mice led towards increased levels of insulin, a sign of insulin resistance, a critical feature of metabolic disorders (MD’S) (21, 22). Additionally, previous studies have also shown that adiposity is exacerbated by IR which is a major contributing risk factor for obesity in metabolic and associated disorders (17, 23). Additionally, we discovered in this study that QUER treatment resulted in a considerable drop in blood insulin levels in PCOS mice, suggesting a potential function for QUER in the treatment of insulin resistance. Patients with PCOS experience hyperandrogenism as well as hyperinsulinemia, which causes adipocytes to increase catecholamine-induced lipolysis, which then results in increased serum free fatty acid levels and dyslipidemia (24). Due to the increased production of free fatty acids by the liver, TG levels in the blood are increased (25). In this work, we discovered that letrozole-treated mice had higher TC and TG levels than the control group. However, the levels significantly dropped after QUER treatment.

Pathological, physiological, and developmental angiogenesis all depend on the angiogenic factor VEGF. Oxidative stress initiates an inflammatory state that, within a feedback cycle, results in both insulin resistance and hyperandrogenism (26). According to reports, PCOS women release more VEGF (27). The mechanism is explained by the fact that the VEGF promoter region contains sites where the androgen receptor (AR) binds. When androgens bind to these locations, the VEGF gene is triggered (28). Additionally, blood of women with PCOS has lower levels of soluble VEGF receptors, which increases the bioavailability of VEGF, as shown by (29). These outcomes are in agreement with the higher VEGF levels seen in this study in the letrozole group, Furthermore VEGF levels were decreased when mice treated with quercetin.

We observed serum hormone levels to confirm the impact of quercetin on hormonal alterations. The most consistent hormonal characteristic of PCOS-affected rats is increased serum levels of androgen and LH/FSH (30), and reduced oestrogen were also noted in PCOS-afflicted mice (17). In this investigation, in contrast to levels in PCOS-afflicted mice, quercetin decreased serum LH/FSH concentration. LH levels above normal and elevated LH and FSH ratio may be used as indicators of PCO among females (31). In addition, quercetin treatment in mice with PCOS significantly reduced serum testosterone levels. PCOS illnesses may benefit from the decreasing of these elevated testosterone levels, since it has already been shown that a high androgen level contributes to aetiology of PCOS (5, 32). Contrary to testosterone, LETZ-treated mice had lower serum oestrogen levels, and the decline was associated with mid or early-follicular growth as well as creation of morphology of follicles in ovary (33). The most successful treatments for atypical symptoms linked to female reproductive illnesses are hormones and chemicals; however, these treatments come with a number of side effects, including uterine haemorrhage, and hyperplasia (34, 35). Such findings imply, quercetin might be an effective medication in treating hormonal imbalances brought on by PCOS. Lower steroid hormone levels in the ovary are correlated with higher numbers of developing follicle’s and different their shapes (36).

*Kitl* and *Bmp1* were used in our work to investigate the ovarian follicle components, and histological investigation was also carried out. In PCOS mice ovaries, the mRNA expression of *Kitl* and *Bmp1*, are declined; and the decline reversed after QUER injection. Histological examination revealed that the PCOS-afflicted mice similarly had many cysts, small follicles, and thin granulosa cell layers. In the past, letrozole-induced follicular dysfunction was also seen, including atretic and large cysts with few granulosa cells (36). Quercetin appears to be involved in the regulation of different parameters linked to follicular development in the ovary because treatment with the drug returned ovarian follicles and its other components in the present investigation, towards normal range.

Quercetin treatment in the current study raised expression of *CYP19a1* in ovary of PCOS-affected mice. Past research has shown that PCOS women have defective aromatase activity, and *CYP19a1* is essential for the normal advancement of the estrous or menstrual phases in PCOS rats (5). Contrarily, reduced aromatase activity in PCOS causes disturbances in oestrogen as well as androgeen generation (37). Here it is demonstrated that mice with PCOS had decreased aromatase activity, which is congruent with the *CYP19a1* and *CYP11a1* mRNA levels. On the other hand, the PCOS plus QUER mice had their aromatase activity restored because *CYP19a1* and *CYP11a1* was highly expressed. Our results on *CYP17A1* expression disagreed with Shah and Patel (38) who reported that quercetin owns useful impact in PCOS via suppressing PI3K that as a result of a reduction in *CYP17A1* gene expression which critically plays function in steroidogenesis process in ovary. Additionally, the mRNA levels of *Ar* and *Esr1* were lowered; however, in our work, quercetin therapy corrected this downregulation in mice with PCOS. *Ar* and *Esr1* transcripts are shown to serve a function of proliferation in folllicular growth (39, 40) and elevated *Ar* levels could facilitate granulosa cell proliferation and differentiation (41). Additionally, quercetin treatment brought back to normal the transcriptional levels of *Fshr* and *Lhr* that had been changed in the ovaries of PCOS mice. *Fshr* moderately controls follicle growth during the baseline follicle growth phase by working in synergy with other stimulating substances like androgens (42). *Lhr* is also present on theca and granulosa cell surfaces, and *Lhr* levels have an impact on ovulation, the development of the corpus lutum, also on synthesis of additional steroids’ such as oestrogen, androgen and progesterone (43). Such findings led us to discover cystic degeneration of the corpus luteum and follicles in PCOS-affected animals. It’s interesting to note that the quercetin helped to slow down the degradation of ovarian follicle development by regenerating the corpus luteum and eliminating cystic follicles. It could be said that quercetinmarkedly reversing ovary physiological functions and regularity of estrous cycle. All these functions have been done via acting of quercetinon pituitary- ovary axis because of reversing normal ration of LH/FSH the main gonadotropic hormones.

Our results agree markedly with Pouteymour et al, (11) and Chen et al. (44) who reviewed Quercetin effects and concluded that it can recover disturbance of ovulation, decrease testosterone and Insulin resistance, adjust metabolism of lipid, ameliorate function of vascular endotheliaum and control intestinal microbiota, that all helps significantly in PCOS’ treatment. Quercetin a bio-flavonoid compound, presents in the glycosylies in vegetables and fruits. Although Quercetin analogs have antioxidant effect attributed to the amount of free “OH” groups in its composition, but Quercetin shows essential hydrophobicity (45). This may cause the recovering effect of Quercetin on PCOS mice in our study. Quercetin medical advantages are attributed to flavanols which serve as ant-inflammatory, antivirus and antioxidative stress, which are considered as three main elements that threaten the health and cause diseases, (11) and (46).

**Figure 8:**
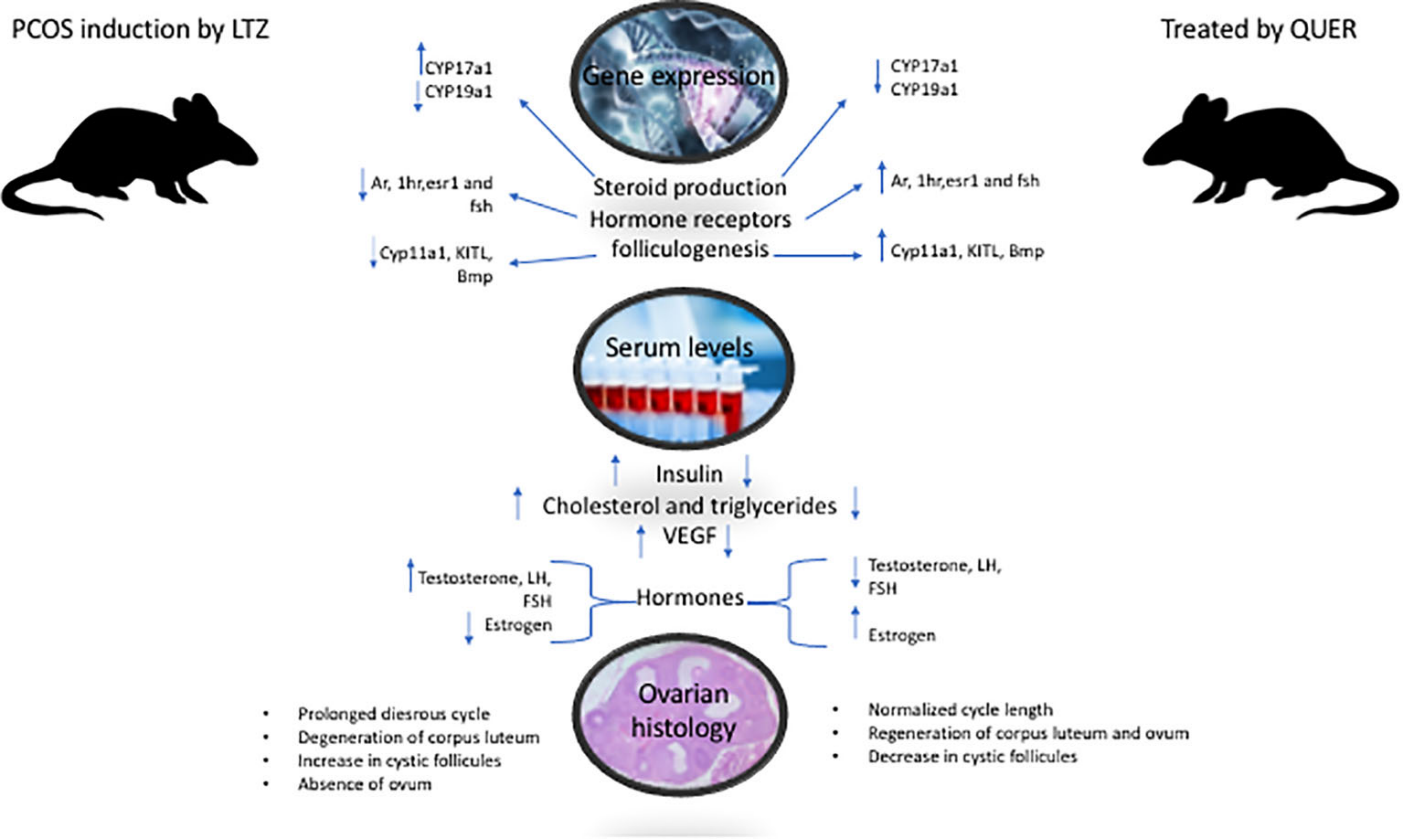
Shows the effect of quercetin on the tested animals in term of Genes expression that control ovary functions. It is observed that quercetin impacted positively by recovering in the PCOS in the ovary by regulating genes expression listed here and restoring hormones and lipid profile.

It is observed that quercetin impacted positively by recovering in the PCOS in different levels: 1) systematically indicated by insulin and lipid profile. 2) Organ level as shown by reserving the ovary histology. 3) Molecular level: it regulated related genes expression and hormones in ovary cells as shown in **Figure 8**.

## 5 Conclusion

This study was designed to illustrate recovering effect of quercetin PCOS induced in mice model. It has proven that quercetin administration reversed experimentally abnormalities in PCOS induced by letrozole. It can speculate that PCOS has modulating- effects on regulatory genes of steroidogenesis and folliculogenesis that leading to the pathophysiological changes include (disturbance in gonadotropin as a result of related gene dysregulation, which followed by regression of ovarian follicles. Interestingly, the study has found that quercetin prevented the degradation of ovarian follicle by regenerating the corpus luteum and eliminating cystic follicles which reset the estrous cycle. The curing action of quercetin on ovary has been seen by recovering gonadotropic and gonads steroids with their physiological functions and regularity of cycle. This indicated markedly that Pituitary- ovary axis was the target of quercetin effect as it reversed normal levels and rations of LH/FSH the main gonadotropic hormones. It worth to mention that quercetin has positively treated PCOS syndrome through different biological aspects: 1) metabolic path: systematically indicated by insulin and lipid profile. 2) histological level as shown by reserving the ovary tissue structure and components. 3) Molecular level: it regulated genes expression listed above. 4) through endocrine mechanism as seen in pituitary and ovary hormones. It could be concluded that quercetin has systematic, histological and molecular improving effects on PCOC. Further research is needed to investigate safety of quercetin on body functions and organs

## Data availability statement

The original contributions presented in the study are included in the article/supplementary materials. Further inquiries can be directed to the corresponding authors.

## Ethics statement

The animal study was reviewed and approved by Institutional ethical committee Barkatullah University Bhopal, India.

## Author contributions

MZUHS: writing original draft, investigation, resources, analysis, methodology. VS: supervision, review and editing. MAM: methodology, software, analysis, review and editing. EAA contributed in scientific editing, drafting final version and revising the manuscript. Rest all authors have reviewed and edited the manuscript. All authors contributed to the article and approved the submitted version.
